# Polyacrylic Acid Hydrogel Coating for Underwater Adhesion: Preparation and Characterization

**DOI:** 10.3390/gels9080616

**Published:** 2023-07-29

**Authors:** Junjie Liu, Nan Hu, Yao Xie, Peng Wang, Jingxiang Chen, Qianhua Kan

**Affiliations:** 1 Applied Mechanics and Structure Safety Key Laboratory of Sichuan Province, School of Mechanics and Aerospace Engineering, Southwest Jiaotong University, Chengdu 610031, China; nanmechanic@163.com (N.H.); wjxxx@my.swjtu.edu.cn (Y.X.); qianhuakan@foxmail.com (Q.K.); 2 School of Aerospace Engineering and Applied Mechanics, Tongji University, Shanghai 200092, China; 3 Facility Design and Instrumentation Institute, China Aerodynamics Research and Development Center, Mianyang 621000, China; jingxiangchen@zju.edu.cn

**Keywords:** hydrogel coating, underwater adhesion, probe-pull test, hydrogen bond, adhesion strength

## Abstract

Underwater adhesion involves bonding substrates in aqueous environments or wet surfaces, with applications in wound dressing, underwater repairs, and underwater soft robotics. In this study, we investigate the underwater adhesion properties of a polyacrylic acid hydrogel coated substrate. The underwater adhesion is facilitated through hydrogen bonds formed at the interface. Our experimental results, obtained through probe-pull tests, demonstrate that the underwater adhesion is rapid and remains unaffected by contact pressure and pH levels ranging from 2.5 to 7.0. However, it shows a slight increase with a larger adhesion area. Additionally, we simulate the debonding process and observe that the high-stress region originates from the outermost bonding region and propagates towards the center, spanning the thickness of the target substrate. Furthermore, we showcase the potential of using the underwater adhesive hydrogel coating to achieve in-situ underwater bonding between a flexible electronic demonstration device and a hydrogel contact lens. This work highlights the advantages of employing hydrogel coatings in underwater adhesion applications and serves as inspiration for the advancement of underwater adhesive hydrogel coatings capable of interacting with a wide range of substrates through diverse chemical and physical interactions at the interface.

## 1. Introduction

Adhesives play a significant role in both everyday life and various industries, offering a wide range of applications [1,2]. There is a vast array of commercially available polymeric adhesives tailored to meet diverse needs and requirements. Typically, adhesives exhibit their effectiveness by establishing either permanent or dynamic bonds with dry, solid surfaces in ambient conditions [3]. Nevertheless, numerous applications necessitate adhesives capable of bonding to wet surfaces or surfaces submerged in aqueous environments [4]. Such requirements arise in various fields, including medical applications like wound dressing and sealing [5,6], underwater repairs [7], underwater assembly of devices [8,9], and underwater soft robotics [10,11]. Hence, the pursuit of adhesive research has become increasingly focused on developing innovative underwater adhesives that exhibit exceptional performance across a wide range of applications within aqueous environments.

Achieving strong adhesion in aqueous environments is more challenging compared to air environments. The presence of a hydration layer on most material surfaces, upon exposure to aqueous environments, impedes molecular-level bridging between the adhesive and the substrate [12]. Furthermore, water drops can become entrapped at the interface between the adhesive and the substrate, leading to a decrease in the effective contact area. Aqueous environments also promote the degradation of underwater adhesion due to the plasticization, hydrolysis, or swelling erosion of the adhesive or the bonding interface. Therefore, developing novel underwater adhesives that can overcome these obstacles and exhibit exceptional performance in aqueous environments is a crucial objective of adhesive research [5,12,13,14,15].

Hydrogels, a class of soft materials, are formed by crosslinked networks of hydrophilic polymers that entrap water molecules, giving rise to their tunable properties and underwater adhesive capabilities [16,17]. Hydrogel-based underwater adhesives mainly have two forms: glue-type and tape-type [12]. The glue-type underwater adhesives behave like a viscoelastic liquid before use that requires a curing process to realize strong underwater adhesion after application. External pressure is usually required to achieve a close intact bond between the target substrate and the adhesive. After the formation of the initial adhesion, the adhesion structure is maintained in an aqueous environment for a period ranging from hours to days in order to allow the curing of the adhesive and the stabilization of the bonding interface. Mussel-inspired catechol chemistry is a popular and promising design strategy to realize underwater adhesion [12,18,19,20,21], because the catechol group can form strong interactions of various types, such as hydrogen bonds, electrostatic interaction, and cation-π interaction, with various substrates in water [15,22,23]. To overcome the hindrance caused by the water film between the adherend and underwater adhesive, water-absorbing fillers or hydrophobic groups have been introduced in these underwater adhesives [24,25]. Glue-type underwater adhesives can also be made via supramolecular assembly from low-molecular-weight molecules [26,27]. Glue-type adhesives are commonly known for their remarkable bonding strength, often surpassing the adhesion strength, and leading to cohesive failure. However, it is important to note that applying such adhesives typically involves longer curing times, allowing the adhesive to fully set and establish a durable bond. Moreover, once the bond is formed, it tends to be irreversible, emphasizing the need for careful alignment and precise application during the bonding process.

Different from glue-type adhesives, tape-type underwater adhesives represent a class of soft solids specifically designed to adhere to substrates in water. As the tape doesn’t require a curing process, underwater adhesion formation using tape-type adhesives is instant [28,29,30]. The strong bonding between the tape and the target substrate can be guaranteed by a molecular interaction or physical suction [12]. To effectively displace the interfacial water between the tape and the target substrate and promote strong molecular interactions, introducing hydrophobic groups or water adsorption components into the tape is a common design strategy [31,32,33]. In addition, the well-designed surface structure of the tape can also lead to the drainage of interfacial water [34,35]. Underwater adhesion realized by physical suction is a controllable and reversible bonding process. This underwater bonding strategy relies on the microstructures of the tape, which provide adhesion via the suction effect accompanied by capillarity [36,37,38]. As the fabrication of the delicate microstructure usually involves a complex process, suction-based underwater adhesive tape is difficult to mass produce.

In this study, we impart the underwater adhesive ability to an inert substrate by applying an underwater adhesive polyacrylic acid (PAAc) hydrogel coating. The PAAc hydrogel coating is prepared using a technique called “hydrogel paint”, which was developed in our previous work [39]. We investigate the underwater adhesion performance of the PAAc hydrogel coating through probe-pull experiments. The effects of contact pressure, adhesion area, ambient pH, and contact time on the strength of underwater adhesion are characterized. The debonding process of the underwater adhesion structure is simulated using the finite element software ABAQUS. Furthermore, we showcase the potential of using the underwater adhesive hydrogel coating to achieve in-situ underwater bonding between a flexible electronics demonstration device and a hydrogel contact lens. Our findings highlight the advantages of employing hydrogel coatings in underwater adhesion applications and serve as inspiration for the advancement of underwater adhesive hydrogel coatings capable of interacting with a wide range of substrates through diverse chemical and physical interactions at the interface.

## 2. Results and Discussion

### 2.1. PAAc Hydrogel Paint

**Figure 1:**
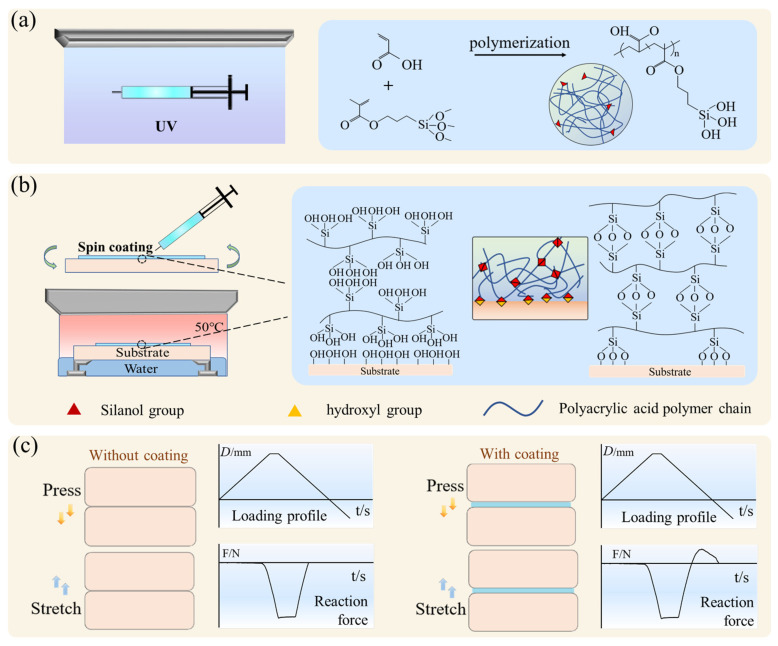
(**a**) PAAc hydrogel paint is prepared by copolymerizing acrylic acid monomer and silane coupling agent under UV exposure. (**b**) PAAc hydrogel paint is applied on the substrate and then incubated in an oven. During the incubation process, the hydrogel paint solidifies and establishes covalent anchors with the substrate. (**c**) In the absence of underwater adhesive hydrogel coating, no adhesion occurs between two contacting substrates. By applying underwater adhesive hydrogel coating to one substrate, underwater adhesion is facilitated.

The PAAc hydrogel paint was prepared via copolymerizing of the acrylic acid (AAc) monomer with the coupling agent 3-(trimethoxysilyl) propyl methacrylate (TMSPMA), as shown in Figure 1a. After applying the PAAc hydrogel paint to a target substrate using a painting technique, e.g., spin coating, the copolymers of the hydrogel paint undergo crosslinking through the condensation of silanol groups, resulting in the gelation of the hydrogel paint (Figure 1b). The condensation rate can be accelerated with increasing temperature. Simultaneously, the silanol groups on the copolymers can undergo condensation with the hydroxyl groups present on the target substrate, forming covalent interfacial bonds between the hydrogel coating and the coated substrate (Figure 1b). These covalent interfacial bonds ensure a strong attachment of the underwater adhesive PAAc hydrogel coating to the substrate. The carboxyl groups on the copolymers are capable of forming hydrogen bonds with various functional groups, including hydroxyl groups, amine groups, and other carboxyl groups. This enables the formation of hydrogen-bonded interlinks between the PAAc hydrogel coating and the target substrate [40]. In the absence of the underwater adhesive hydrogel coating, an inert substrate cannot form a strong bond with a target substrate through simple attachment and compression (the displacement-controlled loading profile and the corresponding reaction force between the two surfaces are depicted in Figure 1c). However, with the assistance of the underwater adhesive hydrogel coating, a robust adhesion is achieved, resulting in an appreciable pull force required to separate the bonded surfaces (depicted in Figure 1c).

### 2.2. Underwater Adhesion Test

**Figure 2:**
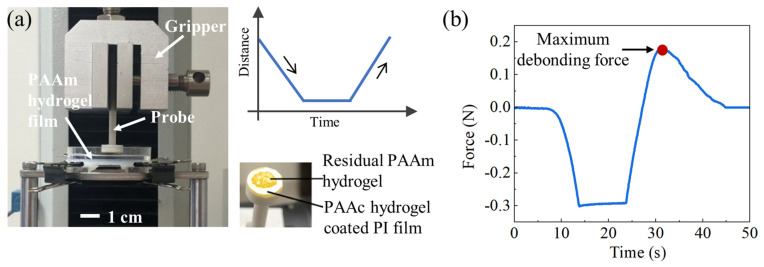
(**a**) Experimental setup and loading profile of a probe for the probe-pull test. Residual PAAm hydrogel on PI film is observed after the probe-pull test. (**b**) Typical force-time relationship for the probe in a loading cycle. Maximum debonding force serves as a measure of the underwater adhesion strength.

The probe-pull test was employed to investigate the adhesion of a PAAc hydrogel-coated substrate to a target substrate underwater. Probes for the tests were fabricated via 3D-printing (BM10-F-250, BLUE MAKER, Shenzhen, China), and the material used for the 3D-printing was polylactic acid (PLA, BLUE MAKER, Shenzhen, China). The 3D-printed probes have a cylindrical handle with a height of 65 mm and a diameter of 5 mm. At one end of the cylindrical handle is a circular plate with a height of 5 mm. Three probes were printed with the circular plate diameter of 10, 12.5, and 15 mm, respectively. As shown in Figure 2a, a PAAc hydrogel-coated PI film was affixed to the bottom surface of the circular plate using super glue (7146, Deli Co. Ltd., Shanghai, China), with the uncoated side serving as the bonding surface. The area of the PAAc hydrogel-coated PI film is identical to the area of the bottom surface of the circular plate. The method used to prepare the PAAc hydrogel coated-PI film can be found in the Section 4. The cylindrical handle of the probe was clamped onto the gripper of the tension test machine (HF-9003S, LIGAO, Shenzhen, China), with the axis parallel to the loading direction.

The target substrate is a polyacrylamide (PAAm) hydrogel with amine groups capable of forming hydrogen bonds with the carboxyl groups in the PAAc hydrogel coating. As a model coated substrate, we chose a polyimide (PI) film with a thickness of 25 μm. PI film is commonly used as a flexible substrate in flexibleelectronics and exhibits stable mechanical properties at high temperatures. Siloxane bonds are responsible for achieving a strong bonding interface between the substrate and the hydrogel coating. Essentially, any substrates that can activate a sufficient number of hydroxyl groups on their surface can form siloxane bonds with the PAAc hydrogel coating [39].

A piece of fully swollen PAAm hydrogel was bonded to the bottom of a Petri dish using super glue. Aqueous solution with various pH levels tuned by acetic acid was added to the Petri dish until it submerged the PAAm hydrogel substrate. The Petri dish with the PAAm hydrogel substrate was securely positioned beneath the gripper of the tension test machine. The surface of the PI film and that of the PAAm hydrogel substrate were kept parallel. The gripper moved towards the PAAm hydrogel substrate at a constant speed of 5 mm/min. Once the pressure between the gripper and the PAAm hydrogel substrate reaches a predetermined value, the probe remains stationary at that location for a while (ranging from 1 s to 10 s) and is then pulled back from the substrate at the same speed as the attachment process. The loading profile for the probe is depicted in Figure 2a. For each experimental condition, a minimum of five probe-pull tests were conducted.

### 2.3. Underwater Adhesion Performance of PAAc Hydrogel Coating

A typical time-force relationship for the probe in a loading cycle is shown in Figure 2b. A PAAc hydrogel coated-PI film is pushed by the probe to attach the PAAm hydrogel substrate. Before contact between the PAAc hydrogel coated-PI film and the PAAm hydrogel substrate is made, the force on the probe is zero. Once they attach, the force on the probe increases with the increasing compression depth due to the elastic deformation of the PAAm hydrogel substrate. When a predefined contact pressure is reached, e.g., 3.82 kPa for the curve in Figure 2b, the probe remains stationary and holds position for a while (ranging from 1 s to 10 s) before being retracted from the PAAm hydrogel substrate. The maximum debonding force in the retraction process divided by the contact area is adapted as a measure of the underwater adhesion strength. The effects of contact pressure, contact size, pH value of the environment, and the contact time on the underwater adhesion strength are investigated through the probe-pull tests. Unless otherwise stated, the contact time, diameter of the contact area, pH value of the environment, and the contact pressure are kept as constants at 5 s, 10 mm, 7, and 3.82 kPa, respectively.

**Figure 3:**
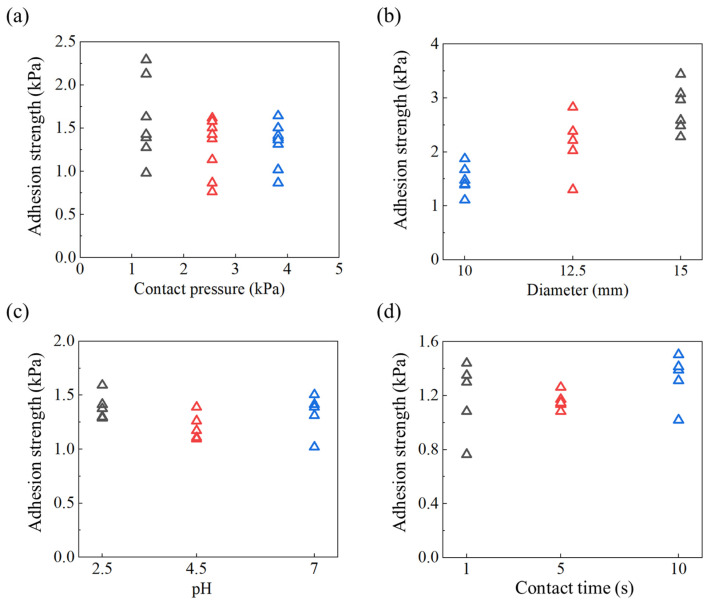
(**a**) Underwater adhesion strength between the PAAc hydrogel coating and the PAAm hydrogel film at various (**a**) contact pressures, (**b**) diameters of contact areas, (**c**) pH levels of environments, and (**d**) contact times.

Figure 3a illustrates the adhesion strength at three different contact pressures: 1.27 kPa, 2.55 kPa, and 3.82 kPa. The adhesion strength remains relatively consistent across the three contact pressures, with the smallest contact pressure resulting in slightly higher adhesion strength than the other two. These findings suggest that even a small contact pressure is sufficient for achieving strong underwater adhesion between the PAAc hydrogel coating and the PAAm hydrogel substrate. However, applying a higher contact pressure could potentially damage the adhesion interface, leading to a slight decrease in the adhesion strength compared to interfaces formed under lower contact pressures.

With an increase in the diameter of the contact area, there is a slight increase in the underwater adhesion strength (Figure 3b). Since the thickness of the PAAm hydrogel substrate remains constant, enlarging the contact area between the PAAc hydrogel coating and the PAAm hydrogel substrate causes a PAAm hydrogel column with a reduced aspect ratio (height divided by diameter) to be stretched upon debonding. Different aspect ratios of a soft bonding system lead to different stress states when the bonding system is separated, resulting in varying bonding strengths. Generally, a large aspect ratio corresponds to a uniaxial stress state, while a small aspect ratio corresponds to a triaxial stress state. The triaxial stress state typically leads to a stronger adhesion strength, as reported in a previous study [41].

In an aqueous environment with pH values of 2.5, 4.5, and 7, the adhesion strength remains nearly constant (Figure 3c). We interpret these results as follows: the interfacial adhesion strength between the PAAm hydrogel substrate and the PAAc hydrogel coating is sufficiently robust to withstand interfacial debonding in all three pH conditions. The debonding primarily occurs through a cohesive fracture of the PAAm hydrogel substrate, and the strength of this substrate is insensitive to the pH value of the environment if that value varies from 2.5 to 7.0. It is worth noting that a pH higher than 7.0 will hinder the formation of the hydrogen bonds at the interface between the PAAc hydrogel coating and target substrate. This, in turn, leads to weak underwater adhesion [40].Consequently, the bonding strength remains dependent on the pH value of the surrounding environment.

The contact time exerts a minimal influence on the underwater adhesion strength (Figure 3d). The establishment of interfacial bonding occurs instantly through the formation of hydrogen bonds at the interface [40]. Prolonging the contact time does not result in enhanced adhesion strength. These results highlight the advantageous characteristics of our underwater adhesive hydrogel coating, which demonstrates rapid adhesion capability to the target substrate in an underwater environment.

### 2.4. Simulation of the Debonding Process

**Figure 4:**
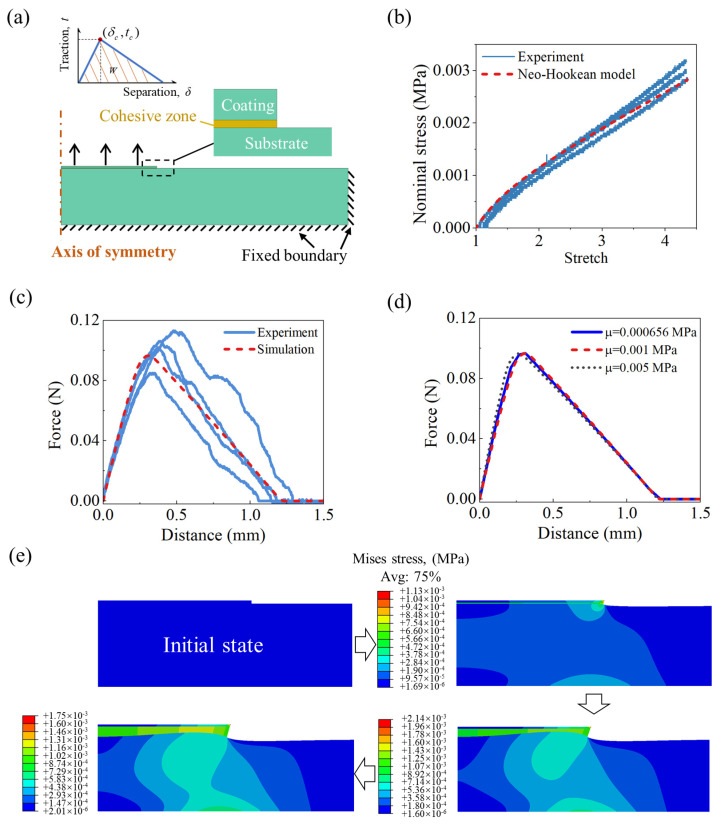
(**a**) Finite element model of the PAAc hydrogel coating-PAAm hydrogel substrate adhesion structure. The adhesion interface is modeled as a cohesive zone. (**b**) Fitting experimental uniaxial stretch-stress curves of PAAm hydrogel substrate by Neo-Hookean model. (**c**) Experiment and simulation distance-force relationship in the debonding process of PAAc hydrogel coating-PAAm hydrogel substrate adhesion structure. (**d**) Distance-force relationships in the debonding process for the PAAc hydrogel coating with various shear modulus. (**e**) Evolution of Mises stress for the PAAc hydrogel coating-PAAm hydrogel substrate adhesion structure in the debonding process.

We conduct a debonding process simulation of the adhesion structure between the PAAc hydrogel coating and the PAAm hydrogel substrate using the finite element software ABAQUS. The finite element model employed is illustrated in Figure 4a. Since the bonding structure is axisymmetric, we simplify the 3-dimensional model into a 2-dimensional model. The PAAm hydrogel substrate has a measured thickness of approximately 2.7 mm, and its radius is three times that of the probe. The bottom and circumferential surfaces of the PAAm hydrogel substrate are fixed, restricting displacement and rotation. Both the PAAm hydrogel substrate and the PAAc hydrogel coating are assumed to be incompressible, and their constitutive behavior is described using the Neo-Hookean model. The Neo-Hookean model equation is given as $W=\frac{\mu }{2}(\lambda_{1}{}^{2}+\lambda_{2}{}^{2}+\lambda_{3}{}^{2}-3)$, where μ is the shear modulus, and $\lambda_{i}$(i=1,2,3) represent the principal stretches, respectively. By fitting the uniaxial stretch-stress curves (Figure 4b), the shear modulus for the PAAm hydrogel substrate is determined to be 0.656 kPa. Since the PAAc hydrogel coating is too thin for a uniaxial tension test, we assign it the same shear modulus as the PAAm hydrogel substrate (0.656 kPa). The subsequent simulation results demonstrate that the shear modulus of the PAAc hydrogel coating has negligible influence on the mechanical response of the adhesion structure in the debonding process.

The PAAc hydrogel coated PI film has a radius of 5 mm, and the thickness of the PAAc hydrogel coating is set to be 100 μm, which is comparable to previous work [39]. In Figure 4a, we have omitted the depiction of the PI film for clarity. Due to the significantly higher stiffness of the PI film compared to the PAAc hydrogel coating and the strong adhesion between them, the lateral displacement and rotation of the top surface of the PAAc hydrogel coating are constrained during the debonding process. The debonding of the PAAc hydrogel coating from the PAAm hydrogel substrate occurs through the applied displacement perpendicular to the bonding interface.

The adhesion interface between the PAAm hydrogel substrate and the PAAc hydrogel coating is represented by a layer of cohesive elements, which exhibit a traction-separation response (Figure 4a). Prior to damage initiation, the cohesive elements behave linearly with a modulus (the slope of the traction-separation curve before damage initiation) matching that of the PAAc hydrogel coating. Damage initiation occurs when the maximum nominal stress reaches the critical value of $t_{c}$. After damage initiation, the cohesive elements undergo progressive damage, leading to a linear decrease in traction and an increasing separation distance. The area under the separation-traction curve represents the energy dissipated per unit area of the cohesive elements during separation. The critical traction force of 0.00132 MPa and the energy *W* of 0.00083 N/mm were obtained from experimental force-distance curves (Figure 4c), which correspond to the debonding of the PAAc hydrogel coating-PAAm hydrogel substrate adhesion structure formed with a contact time of 1 s, contact pressure of 3.82 kPa, a contact area diameter of 10 mm, and pH value of 7. The element type used for both the PAAm hydrogel substrate and the PAAc hydrogel coating is CAX4RH, while for the cohesive elements, the element type is COHAX4.

The simulation results exhibit a satisfactory agreement with the experimental curves (Figure 4c), confirming the validity of the simulation. Additionally, the shear modulus of the PAAc hydrogel coating has a minimal impact on the force-distance relationship during the debonding process (Figure 4d). Figure 4e illustrates the evolution of Mises stress in the adhesion structure between the PAAc hydrogel coating and the PAAm hydrogel substrate during debonding. A region of high stress originates from the outermost adhesion region and propagates towards the center, spanning the thickness of the PAAm hydrogel substrate.

### 2.5. Application of Underwater Adhesive Hydrogel Coating

By employing the underwater adhesive PAAc hydrogel coating approach, we showcase the remarkable underwater adhesion capability of an inert substrate, which requires minimal contact pressure. In the absence of the PAAc hydrogel coating, a bare PI film fails to establish a strong bond with a PAAm hydrogel in an aqueous environment (see Video S1). However, when the PI film is coated with the PAAc hydrogel, it adheres firmly to a submerged PAAm hydrogel substrate (see Video S2). This bonding structure withstands bending without any noticeable signs of debonding (see Video S3), and the bonded PAAm hydrogel exhibits resistance to detachment from the PI film.

**Figure 5:**
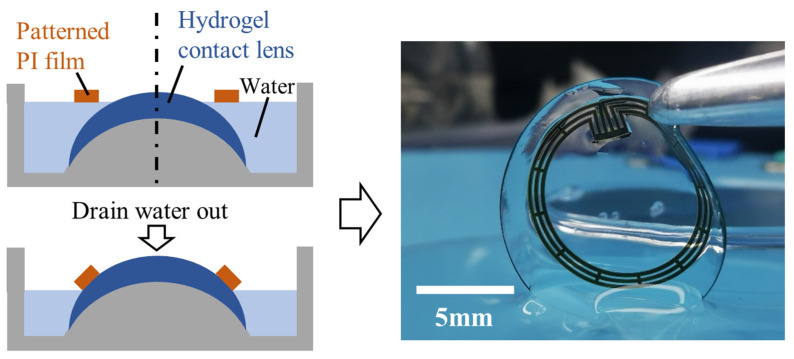
Schematic of the assembly process of the hydrogel contact lens and patterned PI film. Strong adhesion forms between the PI film and hydrogel contact lens through underwater adhesive PAAc hydrogel coating.

The underwater adhesive PAAc hydrogel coating holds great potential for in-situ bonding between flexible electronics and soft materials. Since both flexible electronics and soft materials are delicate and pliable, achieving in-situ assembly and forming reliable interfacial bonding between them can be challenging. To demonstrate the capabilities of hydrogel coating in addressing this issue, we utilize a commercial hydrogel contact lens (4978394, Bausch and Lomb Incorporated, Vaughan, ON, Canada) as a representative soft substrate and a patterned PI film as a flexible electronics demo device. A specially designed mold, depicted in Figure 5, is employed for the in-situ assembly process. The mold contains DI water, with the hydrogel contact lens placed at the bottom. The patterned PI film floats on the surface of the water. By gradually draining the water from the mold, the water level decreases, bringing the patterned PI film into contact with the hydrogel contact lens. Thanks to the underwater adhesive PAAc hydrogel coating on the PI film, strong underwater adhesion occurs instantaneously upon contact with the contact lens (Figure 5).

## 3. Conclusions

In this study, we demonstrate that the underwater adhesive capability can be imparted to an inert substrate through a PAAc hydrogel coating, which forms hydrogen bonds with a target substrate at the adhesion interface. The formation of the underwater adhesion is rapid, occurring within one second, and remains unaffected by the pH value of the environment (ranging from 2.5 to 7.0) and contact pressure (ranging from 1.27 kPa to 3.82 kPa). Additionally, the adhesion strength slightly increases with an enlarged adhesion area. We simulated the debonding process of the PAAc hydrogel coating-PAAm hydrogel substrate adhesion structure, revealing the initiation of high-stress regions from the outermost adhesion region that propagate towards the center, spanning the thickness of the PAAm hydrogel substrate. The underwater adhesive PAAc hydrogel coating exhibits promising potential for in-situ bonding between flexible electronics and soft materials. Designing underwater adhesive hydrogel coatings capable of interacting with a wide range of substrates through diverse chemical and physical interactions at the interface is worthy of further study.

## 4. Materials and Methods

### 4.1. Materials

Acrylic acid (AAc, 80001418), acrylamide (AAm, 800013290), N,N′-Methylenebis (acrylamide) (MBAA, 30117826), potassium persulfate (KPS, 10017418), (3-mercaptppropyl) trimethoxysilane (CTA, XW010044), N,N,N′,N′-tetramethylethylenediamine (TMEDA, 30117826), and ethyl alcohol (10009128) were purchased from the Sinopharm Chemical Reagent Co., Ltd. (Shanghai, China). 3-(trimethoxysilyl) propyl methacrylate (TMSPMA, W320005) and acetic acid (AA, B020052) were purchased from the Energy Chemical Co., Ltd. (Shanghai, China). All chemicals were purchased and used without further purification. Deionized (DI) water was purchased from Wahaha Co., Ltd. (Hangzhou, China). Polyimide film (Kapton, 100HN) with a thickness of 0.025 mm was purchased from Dupont (Wilmington, NC, USA).

### 4.2. Synthsis of the PAAc Hydrogel Paint

For every 1 mL AAc solution with a concentration of 2 M (2 moles AAc dissolved in 1 L of DI water), 40 μL KPS solution with a concentration of 0.1 M, 2 μL TMSPMA, 10 μL TMEDA solution with 10% volume ratio in DI water, and 10 μL CTA solution with 10% volume ratio in ethyl alcohol were added, followed by vortex mixing (MX-S, DLAB, Beijing, China) for 30 s. The resulting solution was drawn into a plastic syringe (5 mL, KDL, Shanghai, China) and subject to UV irradiation (18 W, ZIGU, Beijing, China, 6.5 cm distance between sample and bulb). After 50 min, 5 mL of the PAAc hydrogel paint was obtained.

### 4.3. Synthsis of the PAAm Hydrogel Substrate

The acrylic mold used for synthesizing the PAAm hydrogel substrate consists of two parts: base plate and covering plate. The base plate was made by bonding an acrylic ring with thickness of 2 mm, inner diameter of 80 mm, and outer diameter of 120 mm to a circular acrylic plate with diameter of 120 mm and thickness of 2 mm. The adhesive used is the super glue (7146, Deli Co., Ltd., Shanghai, China). The covering plate is a circular acrylic plate with a thickness of 2 mm and diameter of 120 mm. The surface of the mold was not treated further.

For every 1 mL AAm solution with a concentration of 2 M (2 moles AAm dissolved in 1 L of DI water), 10 μL MBAA solution with a concentration of 0.1 M, 50 μL KPS solution with a concentration of 0.1 M, and 5 μL TMEDA solution with 10% volume ratio in DI water were added. 20 mL of the above solution was vortex mixed for 30 s and the obtained precursor solution was poured into the base plate of the mold. The added solution slightly surpasses the capacity of the base plate. After covering the solution-filled base plate with the covering plate, the extra solution was expelled from the mold and the solution filled the space between the base plate and covering plate without visible air bubbles. Next, the mold was clamped (1091606, Deli Co., Ltd., Shanghai, China) and placed at room temperature for 24 h to undergo hydrogel precursor solidification. Finally, a cured PAAm hydrogel membrane with a diameter of 80 mm and thickness of 2 mm was obtained. We repeated the experiment five times and got five pieces of the PAAm hydrogel membrane. Before conducting the underwater adhesion test, the PAAm hydrogel membrane was fully swelled in DI water for at least 24 h.

### 4.4. Preparation of the PAAc Hydrogel Coated PI Film

A piece of PI film with a thickness of 25 μm and a width and length of 60 mm was fixed on a glass plate with a width and length of 80 mm and a thickness of 2 mm using tape (1838104, Deli Co., Ltd., Shanghai, China). The PI film-bonded glass plate was placed into a plasma treatment machine (TS-PL02, Tonson Tech, Shenzhen, China) for treating for 90 s to generate hydroxyl groups on the PI surface. After the plasma treatment, the PI film-bonded glass plate was immediately fixed on the rotation plate of a spin coater (KW-4A, REEDEA, Beijing, China) through negative pressure. Next, we added about 0.5 mL of the prepared PAAc hydrogel paint to the center of the plasma treated PI film. The PAAc hydrogel paint was spin coated on the PI film at a speed of 1000 r/min for 45 s. Finally, we placed the PI film-bonded glass plate in an oven (101-00B, SHANGYI, Shanghai, China) with a temperature of 50 °C for 1 h to facilitate curing of the PAAc hydrogel coating and the bonding formation between the PAAc hydrogel and the PI film. A PAAc hydrogel coated PI film was obtained by debonding the PI film from the glass plate.

### 4.5. Uniaxial Tension Test

The fully swelled PAAm hydrogel substrate was cut into slices with a width of 10 mm and a thickness of 2.7 mm. The gauge length of the PAAm hydrogel slice loaded on a uniaxial tension test machine (HF-9003S, LIGAO, Shenzhen, China) was 60 mm. The stretch speed applied on the PAAm hydrogel slice was 12 mm/min. Three PAAm hydrogel samples were made and tested.

## Data Availability

The data are available from the corresponding author, J. Liu, upon reasonable request.

## Supplementary Materials

The following supporting information can be downloaded at: https://www.mdpi.com/article/10.3390/gels9080616/s1, Video S1: Underwater adhesion between a bare PI film and a PAAm hydrogel substrate; Video S2: Underwater adhesion between a PAAc-hydrogel-coated PI film and a PAAm hydrogel substrate; Video S3: Bending the bonding structure between the PAAc-hydrogel-coated PI film and the PAAm hydrogel substrate; Video S4: Peeling the PAAm hydrogel off the bonding structure between the PAAc-hydrogel-coated PI film and the PAAm hydrogel substrate
